# A pig multi-tissue normalised cDNA library: large-scale sequencing, cluster analysis and 9K micro-array resource generation

**DOI:** 10.1186/1471-2164-9-17

**Published:** 2008-01-14

**Authors:** Agnès Bonnet, Eddie Iannuccelli, Karine Hugot, Francis Benne, Maria F Bonaldo, Marcelo B Soares, François Hatey, Gwenola Tosser-Klopp

**Affiliations:** 1 Laboratoire de Génétique Cellulaire, INRA, UMR444, Institut National de la Recherche Agronomique, F-31326 Castanet-Tolosan, France; 2 Sigenae, INRA, Institut National de la Recherche Agronomique, F-31326 Castanet-Tolosan, France; 3 Laboratoire de Radiobiologie et d'Etude du Génome, UMR314, INRA, CRB GADIE, Institut National de la Recherche Agronomique, F-78352 Jouy-en-Josas, France; 4 Laboratoire de Radiobiologie et d'Etude du Génome, CEA, DSV, IRCM, Commissariat à l'Energie Atomique, F-78352 Jouy-en-Josas, France; 5 Children's Memorial Research Center, Northwestern University's Feinberg School of Medicine, Chicago, IL, USA

## Abstract

**Background:**

Domestic animal breeding and product quality improvement require the control of reproduction, nutrition, health and welfare in these animals. It is thus necessary to improve our knowledge of the major physiological functions and their interactions. This would be greatly enhanced by the availability of expressed gene sequences in the databases and by cDNA arrays allowing the transcriptome analysis of any function.

The objective within the AGENAE French program was to initiate a high-throughput cDNA sequencing program of a 38-tissue normalised library and generate a diverse microarray for transcriptome analysis in pig species.

**Results:**

We constructed a multi-tissue cDNA library, which was normalised and subtracted to reduce the redundancy of the clones. Expressed Sequence Tags were produced and 24449 high-quality sequences were released in EMBL database. The assembly of all the public ESTs (available through SIGENAE website) resulted in 40786 contigs and 54653 singletons. At least one Agenae sequence is present in 11969 contigs (12.5%) and in 9291 of the deeper-than-one-contigs (22.8%). Sequence analysis showed that both normalisation and subtraction processes were successful and that the initial tissue complexity was maintained in the final libraries. A 9K nylon cDNA microarray was produced and is available through CRB-GADIE. It will allow high sensitivity transcriptome analyses in pigs.

**Conclusion:**

In the present work, a pig multi-tissue cDNA library was constructed and a 9K cDNA microarray designed. It contributes to the Expressed Sequence Tags pig data, and offers a valuable tool for transcriptome analysis.

## Background

In pigs, like in other domestic animals, breeding and product quality improvement require the control of several different traits (reproduction, nutrition, health and welfare). It is thus necessary to improve our knowledge of the major physiological functions and their interactions. For this purpose, the French National Institute for Agricultural Research (INRA) [1] has launched a genomic research program, AGENAE (Analyse du GENome des Animaux d'Elevage) [2] for the identification and the functional and genetic characterisation of a large number of genes in cattle, pigs, chicken and trout [3].

With the shift from map-based towards sequence-based gene discovery, the prevailing approach for creating transcription maps has become the generation of Expressed Sequence Tags [4]. In pigs, the first EST project [5] and first large-scale EST project were reported [6] about ten years ago. Subsequently, several research groups have generated ESTs from cDNA libraries constructed from either a single porcine tissue or a limited number of tissues related to a stage of development or a function, such as anterior pituitary [7,8], backfat [9], brain [10], liver [11], skeletal muscle [12-14], immune system tissues [15], reproductive tissues [8,16,17] and embryo [17-19]. The construction of full-length cDNA libraries was reported more recently [20-22].

To date, the construction of several pig arrays have been reported. Some of them, with various supports, contain 1 to 4000 cDNA from specific libraries: brain tissue[10] (GEO database accession number GPL336), muscle [23] (GPL518)[24] (GPL2731), embryo [25] (GPL1209), immune system cells [26,27] (GPL1624), but others aim at a generic analysis (10 to 20000 genes) of pig transcriptome with glass slides of in situ-synthesised oligonucleotides (Affymetrix, GPL3533), spotted oligonucleotides (Operon-Qiagen set) (GPL 1881, GPL3461, GPL3707) or cDNAs (GPL3585, GPL3608).

We report here the construction of a pig multi-tissue cDNA library, its sequencing and analysis, and the generation of a 9K nylon micro-array public tool for large scale expression profiling experiments.

## Results and Discussion

### cDNA libraries construction and characterisation

Starting from 38 tissues, six initial libraries containing 780 000 to 1800 000 recombinant clones were generated (Table 1). Their average insert size was 1.2 kb. The pooling and normalisation led to a 6.4 million-clone library and the sub-library of abundant clones contained 700000 clones. The average insert length of the normalised library was 1 kb and and the proportion of empty clones was low (2%).

**Table 1:** Description of the different libraries

N° library	Tissues Adult (A), young (Y) or fetal (F) animal	Number of recombinant clones
1-Brain	Hippocampus (A)	800 000
	Hypothalamus (A)	
	Pituitary gland (A)	
	Cerebral trunk (A)	
	Brain (F)	
2-Digestive function	Stomach (A + F)	822 500
	Small intestine (A + F)	
	Large intestine (A + F)	
	Gall-bladder (A)	
3-Glands	Adrenals (A)	800 000
	Kidney (A)	
	Liver (A + F)	
	Thymus (A + Y)	
	Spleen (A)	
	Pancreas (A)	
4-Heart and muscle	Heart (A + F)	1 800 000
	Muscle (A + F)	
	Skin (A)	
	Melanocytes (A)	
	Adipose tissue (A)	
5-Male reproductive organs	Gonads (F)	780 000
	Epididymis (A)	
	Seminal vesicle (A)	
	Bulbourethral gland (A)	
	Testis (A)	
6-Female reproductive organs	Gonads (F)	1 325 000
	Ovary (A + F)	
	Uterus (A)	
	Placenta	
	Mammary gland (A)	
Normalized (N)	Mix of libraries 1 to 6	6 400 000

Tissue samples from Meishan and Large White pigs at different stages of development or in different physiological conditions (fetus (F), young (Y) or adult animal (A), pregnant, stressed or control animals) were taken and 6 libraries were constructed.

**Figure 1:**
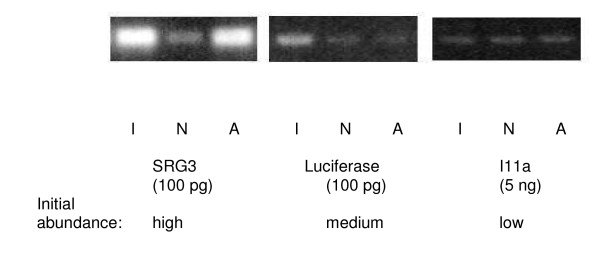
**Control of the normalisation procedure**. Normalisation process efficiency was tested by using specific amplification of the control genes SRG3, Luciferase and I11a. Thirty cycles of amplification have been performed, using indicated amounts of plasmid DNA from the initial library (I), the normalised library (N), or the library of abundant clones (A). The frequency of the controls in the initial library is indicated.

PCR amplification with specific primers for the external control genes SRG3, luciferase and I11a (abundant, medium and low-frequency) was used to check the normalisation process (Figure 1). Southern blot experiments demonstrated that the abundance of actin gene and of the abundant spike mRNA SRG3 have been greatly reduced by the normalisation process (data not shown). In addition, quantitative PCR experiments (data not shown) demonstrated that the representation of SRG3 had been reduced 5800 times, the representation of luciferase reduced 4 times and the representation of I11a increased 1.5 times. In the normalised library, the representation of the external controls was estimated as follows: SRG3 = 0.0009%, luciferase = 0.0125%, I11a = 0.0008% as compared to the initial frequencies of 10%, 0.1% and 0.001% respectively.

After a first round of sequencing, the library was subtracted with the 8736 already-sequenced clones. The subtracted library contained 60 000 clones. The quality of the subtraction was assessed by the sequencing of 384 clones (see below).

### Sequencing

**Figure 2:**
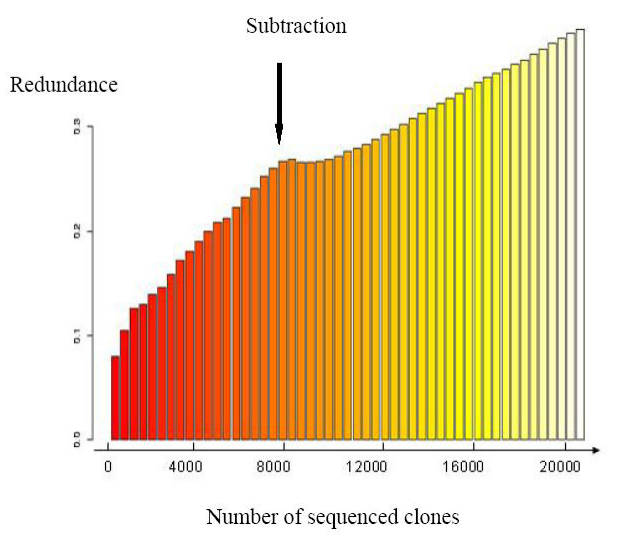
**Evolution of redundancy rate**. Redundancy of the library was calculated as follows: redundance = 1 - (number of genes/number of clones). The number of genes is estimated by the number of contigs obtained at the end of the SIGENAE processing chain.

High-throughput sequencing was carried out on the normalised library. Sequencing was performed from both ends for 5664 clones. The sequencing effort was continued from 5'-end only for the next 3072 clones. A total of 14400 sequences were generated from the multi-tissue normalised library and 11671 valid sequences (81.7%) were submitted to EMBL-EBI nucleotide database (Table 2, Additional file 1) [28]. PolyA was detected in 19.7% of the 5'-end sequences and 67.6% of the 3'-end sequences. Polyadenylation signal was detected in 59.6% of the polyA-containing sequences with 51.4% of AAUAAA signal, which is consistent with previous estimations in humans [29]. The sequence of the medium-frequency external control (luciferase) was present 7 times (0.06%), which is higher than the estimated representation of the luciferase control (0.0125%) in the normalised library. The other two external control sequences were not detected (<0.0085%), which is consistent with the estimated frequencies of these 2 controls (0.0008 and 0.0009%). The proportion of fully-sequenced clones was 36.8% for both-ends sequenced clones and 12.9% for 5'-end sequenced clones. At this stage, the redundancy rate of the sequences had reached 25.5% (Figure 2). One contig, corresponding to 367 clones out of 8736 (4.2%) was obviously responsible for a high proportion of redundancy. It corresponded to a 28S RNA contamination, which was over-representated (10%) by 4 members of the 28S RNA contig in the driver, during subtraction of the normalised library.

**Table 2:** Number of sequenced and released ESTs from the two Agenae libraries

Libraries	Normalised	Subtracted	Total
Number of sequenced clones	8736	14976	23712
Number of 5' sequences	8736	14976	23712
Number of 3' sequences	5664	0	5664
Number of sequences	14400	14976	29376

Published sequences	11671	12778	24449

In the first 384-sequences from the subtracted library, the proportion of 28S RNA sequences had decreased towards 0.52% and 95.1% sequences were new, in comparison with the normalised library. The proportion of empty clones was still about 2%. A total of 14976 clones were then 5'-end sequenced and 12778 (85.3%) sequences were released in the EMBL-EBI nucleotide database (Table 2) [28]. Sequencing was then stopped: the redundancy had reached 39% (Figure 2). The EMBL accession numbers are listed in supplemental data 1. PolyA was detected in 41.5% of the sequences. Polyadenylation signal was detected in 52.9% of the polyA-containing sequences. The sequence of the medium-frequency external control (luciferase) was present 5 times. The other two external control sequences were still not detected.

The library construction method (through the excess of oligo(dT) during the first reverse transcription) led to short polyA 3'-end stretches, allowing almost the same validity rate of the sequences either from 5' (82.2%) or 3' (80.9%)-end. Thus, even if 3'-end sequencing is useful to distinguish genes in a closely related family as the 3'-end non coding regions are more divergent, the 5'-end sequencing strategy was favoured to provide better annotated clones.

### Sequence assembly and analysis

#### Agenae contribution to public sequence data

**Figure 3:**
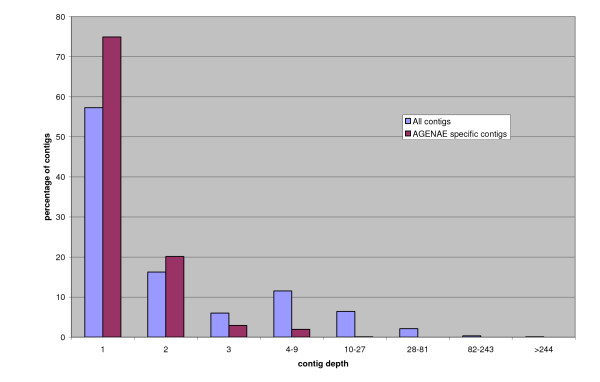
**Histogram of contig depths**. The proportion of Agenae-specific contigs (purple squares) and public porcine contigs (blue squares) is represented in the different contig size classes.

Clustering of the 437,656 public pig sequences, including ours, resulted in 40,786 contigs and 54,653 singletons (psc3 clustering version [30]). Agenae sequences represent 4.9% of the published sequences. At least one Agenae sequence is present in 11,969 contigs (12.5%) and in 9,291 of the deeper-than-one-contigs (22.8%). The assembly shows that 3574 contigs are specific of the AGENAE library. A high proportion of these contigs are singletons (75%), which is higher than the proportion of singletons in the whole-data porcine assembly (52%). This observation and the absence of high-depth specific contigs are evidence of the good normalisation and subtraction processes (Figure 3). Although Agenae sequences are a relatively small contribution to the international sequencing effort, they offered a very good tool to design cDNA microarrays, since they represented 22.8% of the deeper-than-one contigs. The cDNA clones are also a valuable tool for gene expression studies.

The number of contigs highly depends on the parameters used when assembling the sequences. The TIGR pig clustering[31], with nearly the same amount of data, lead to 64,746 contigs and 88,274 singletons. Careful annotation of the contigs and the next completion of the pig genome sequence may show that paralogous genes are sometimes clustered and that, on the contrary, overlapping contigs may have been split up. UniGene clustering [32] lead to 32,711 contigs and 7,230 singletons. The low number of singletons in UniGene assembly is probably due to the fact UniGene does not use part of the available singletons, as was already noticed with trout data [3].

#### Quality of the libraries

The analysis of the twenty deepest contigs (Table 3) shows a high representation of ribosomal proteins, serum proteins, translation factor, that are often over-represented in cDNA libraries [33,34]. Eighteen of these twenty contigs are represented by at least one AGENAE EST. However, the average frequency of AGENAE ESTs for these 18 contigs is 0.5%, which is about ten times less than the frequency of AGENAE EST in the public databases (4.9%). This shows, again, that the normalisation and subtraction processes were efficient.

**Table 3:** 20 deepest contigs

Name	contig depth	%AGENAE EST	Best swissprot hit	Best SP hit accession	Best SP hit description	Best SP hit evalue
BM658630.1.p.sc.3	2448	0.1	P68363	TBAK_HUMAN	Tubulin alpha-ubiquitous chain (Alpha-tubulin ubiquitous) (Tubulin K-alpha-1)	0
BM194705.1.p.sc.3	1945	0.1	P68105	EF1A1_RABIT	Elongation factor 1-alpha 1 (EF-1-alpha-1)	0
BM658885.1.p.sc.3	1656	0.2	Q6QRN9	ADT3_PIG	ADP/ATP translocase 3 (Adenine nucleotide translocator 2) (ANT 3)	1.00E-161
BM484007.1.p.sc.3	1533	0.1	Q5R536	AACT_PONPY	Alpha-1-antichymotrypsin precursor (ACT)	1.00E-126
C94874.1.p.sc.3	1408	0.2	P48819	VTNC_PIG	Vitronectin precursor (Serum spreading factor) (S-protein)	0
AJ275280.1.p.sc.3	1278	0.5	O46415	FRIL_BOVIN	Ferritin light chain (Ferritin L subunit)	4.00E-86
BM658563.1.p.sc.3	1257	0.4	P63245	GBLP_RAT	Guanine nucleotide-binding protein beta subunit 2-like 1	0
BM083203.1.p.sc.3	1246	0.8	P61288	TCTP_PIG	Translationally-controlled tumor protein (TCTP)	5.00E-96
BQ598787.1.p.sc.3	1147	0.1	P63221	RS21_PIG	40S ribosomal protein S21	2.00E-41
BM658711.1.p.sc.3	1096	0.3	P05388	RLA0_HUMAN	60S acidic ribosomal protein P0 (L10E)	1.00E-145
BM190112.1.p.sc.3	1045	0.2	P08267	FRIH_CHICK	Ferritin heavy chain (EC 1.16.3.1) (Ferritin H subunit)	4.00E-95
BM659089.1.p.sc.3	1035	0.2	P02672	FIBA_BOVIN	Fibrinogen alpha chain [Contains: Fibrinopeptide A] (Fragment)	0
BM190048.1.p.sc.3	1021	2.4	P01965	HBA_PIG	Hemoglobin alpha subunit (Hemoglobin alpha chain) (Alpha-globin)	4.00E-77
BM659181.1.p.sc.3	838	0.6	Q8SPS7	HPT_PIG	Haptoglobin precursor [Contains: Haptoglobin alpha chain; Haptoglobin beta chain]	0
BM659099.1.p.sc.3	825	1.6	P08835	ALBU_PIG	Serum albumin precursor	0
CF360997.1.p.sc.3	779	0.5	O46658	CP2DP_PIG	Cytochrome P450 2D25 (EC 1.14.14.-) (CYPIID25) (Vitamin D(3) 25-hydroxylase)	0
BQ598755.1.p.sc.3	769	0.4	Q29387	EF1G_PIG	Elongation factor 1-gamma (EF-1-gamma) (eEF-1B gamma) (Fragment)	0
CF359328.1.p.sc.3	711	0.8	Q8WNV7	DHRS4_PIG	Dehydrogenase/reductase SDR family member 4 (EC 1.1.1.184)	1.00E-129
BQ598401.1.p.sc.3	672	0.0	P39872	RL3_BOVIN	60S ribosomal protein L3	0
BQ604206.1.p.sc.3	660	0.0	P01903	2DRA_HUMAN	HLA class II histocompatibility antigen, DR alpha chain precursor	1.00E-103

The 20 deepest contigs from all public pig cDNA libraries were listed with their Sigenae contig name, depth, % of Agenae ESTs, Best Swissprot hit, hit accession, hit description and evalue, as annotated in the Sigenae web interface [30].

The analysis of the sequences obtained from the normalised library revealed a contamination by 28S ribosomal RNA This type of contamination has already been described in cDNA libraries [35]. This sequence has been over-represented in the subtraction driver and the analysis of the sequences from the subtracted library reveals the presence of 127 out of 14,976 28S ribosomal clones (0.85%). The proportion of this contamination has then been reduced by about 5.

The twenty deepest AGENAE-specific-contigs are listed in Table 4. No hit is found with pig proteins. The best swissprot hit of 16 contigs are with either primates (human, or chimpanzee) rodent (mouse, rat) or other mammals (dog, bovine). Four contigs do not have any swissprot hit. As many tissues were mixed to construct the libraries, without tagging of the cDNAs, specific-tissue sequences were searched. The results are listed in Table 5. The TrainA protein, which is only expressed in epididymis is found [36]. So are GDF9, which is specific to ovary and over-expressed in oocytes [37] and specific mRNA for heart, pituitary gland, muscle or stomach, demonstrating that the multi-tissue library strategy was efficient to get low-redundancy information from a large set of tissues.

**Table 4:** 20 deepest Agenae specific contigs

Name	contig depth	Best swissprot hit	Best SP hit accession	Best SP hit description	Best SP hit e-value
BX666702.1.p.sc.3	18	P04813	CTR2_CANFA	Chymotrypsinogen 2 precursor (EC 3.4.21.1)	1.00E-108
BX666408.1.p.sc.3	16	P08723	SPBP_RAT	Prostatic spermine-binding protein precursor (SBP)	7.00E-16
BX914621.1.p.sc.3	11	Q01167	FOXK2_HUMAN	Forkhead box protein K2 (Interleukin enhancer-binding factor 1)	0
BX671136.1.p.sc.3	9	Q29463	TRY2_BOVIN	Anionic trypsin precursor (EC 3.4.21.4)	1.00E-126
BX914474.1.p.sc.3	9	Q76G19	PDZK4_HUMAN	PDZ domain-containing protein 4	2.00E-81
BX666344.1.p.sc.3	8	Q9D269	CST11_MOUSE	Cystatin-11 precursor	1.00E-34
BX668876.1.p.sc.3	8	O75376	NCOR1_HUMAN	Nuclear receptor corepressor 1 (N-CoR1) (N-CoR)	0
BX917545.1.p.sc.3	8	NONE	NONE	NONE	
BX669605.1.p.sc.3	7	Q9P1Z0	ZBTB4_HUMAN	Zinc finger and BTB domain-containing protein 4	6.00E-54
BX670760.1.p.sc.3	7	P19835	CEL_HUMAN	Bile-salt-activated lipase precursor (EC 3.1.1.3)	1.00E-133
BX914935.1.p.sc.3	7	Q96NJ5	BKLH5_HUMAN	BTB and kelch domain containing protein 5	2.00E-17
BX915192.1.p.sc.3	7	NONE	NONE	NONE	
BX916737.1.p.sc.3	7	P51611	HCFC1_MESAU	Host cell factor (HCF) (HCF-1) (C1 factor) (VP16 accessory protein)	6.00E-85
BX665022.1.p.sc.3	6	Q5R7B5	KCRS_PONPY	Creatine kinase, sarcomeric mitochondrial precursor (EC 2.7.3.2)	0
BX665363.1.p.sc.3	6	Q9NST1	ADPN_HUMAN	Adiponutrin (iPLA2-epsilon) [Includes: Triacylglycerol lipase (EC 3.1.1.3)	3.00E-24
BX665415.1.p.sc.3	6	Q13516	OLIG2_HUMAN	Oligodendrocyte transcription factor 2 (Oligo2) (Basic helix-loop-helix protein class B 1)	8.00E-65
BX670113.1.p.sc.3	6	NONE	NONE	NONE	
BX675854.1.p.sc.3	6	O75376	NCOR1_HUMAN	Nuclear receptor corepressor 1 (N-CoR1) (N-CoR)	7.00E-71
BX667060.1.p.sc.3	5	NONE	NONE	NONE	
BX914492.1.p.sc.3	5	Q30KL7	DB109_PANTR	Beta-defensin 109 precursor (Defensin, beta 109)	4.00E-27

The 20 deepest contigs containing only Agenae pig ESTs were listed with their Sigenae contig name, depth, Best Swissprot hit, hit accession, hit description and e-value, as annotated in the Sigenae web interface [30].

**Table 5:** tissue-specific contigs

Library number	Tissue	Best swissprot hit	e-value	Species	Reference	Clone	Genbank accession number	Sigenae contig name	Contig depth
1	pituitary gland	FSHB_PIG (P01228)	5.00E-78	Pig		scan0030.f.01	BX916158	BQ597499.1.p.sc.3	26
2	stomach	MUC5A_HUMAN (P98088)	0.00E+00	Human	[46]	scac0042.l.09	BX674468	BX674468.1.p.sc.3	3
3–4	muscle/liver	MYOZ1_PIG (Q4PS85)	1.00E-132	Pig	[47]	scac0038.p.22	BX673406	CF179827.1.p.sc.3	23
4	heart	DNJA4_HUMAN (Q8WW22)	0	Human	[48]	scan0007.c.04	BX919910	BM190198.1.p.sc.3	11
4	muscle/heart	MYOZ2_PONPY (Q5R6I2)	1.00E-121	Orangutan	[47]	scac0043.l.05	BX676752	BM189987.1.p.sc.3	12
4	muscle	CAV3_HUMAN (P56539)	1.00E-81	Human	[49]	scac0041.p.13	BX672700	BX672700.1.p.sc.3	5
5	epididymis	RNAS1_RAT (P00684)	1.00E-08	rat	[36]	scan0009.m.06	BX919901	BX664890.1.p.sc.3	23
5	epididymis	GPX5_PIG (O18994)	1.00E-129	Pig	[50]	scan0028.l.11	BX914773	BX914773.1.p.sc.3	6
6	ovary	GDF9_SHEEP (O77681)	0	sheep	[37]	scac0039.l.15	BX675058	BX671944.1.p.sc.3	13

For each initial libray, at least one mammalian sequence of tissue-specific mRNAs (identified in the literature) was blasted against sigenae contigs. The Sigenae contig name with a significant blast e-value is in the table, with its depth and one Agenae clone and sequence.

### Microarray design and production

#### Design

Among the 95439 SIGENAE pig contigs, 8931 different contigs were chosen. For 7749 of them, at least one representing clone belonged to the multi-tissue library and had an insert size compatible with PCR amplification (data not shown). Other contigs were either represented by a USDA clone (835) [17] or a subtractive suppression library clone (188) (Agnès Bonnet, personal communication). Other clones come from different home-made libraries (159) and 285 controls were also included (78 empty controls, 12 empty-vector controls and 195 spikes).

#### Microarray quality control

**Figure 4:**
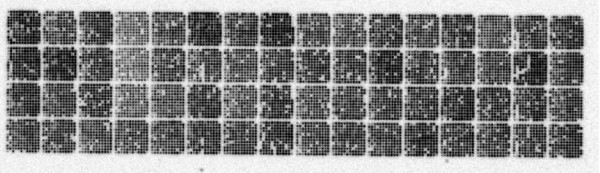
**Hybridisation of the generic 9K pig microarray with an oligonucleotide probe**. The array is composed of 64 (16*4) blocks of 144 (12*12) spots. At the four corners of each block, 3 external controls and a negative control (upper right corner) are spotted.

The successive steps in the microarrays production have been checked for quality. Firstly, the rearraying of the 9216 selected clones or controls has been checked by sequencing 4 clones from each plate corner. A conformity of 97.5% has been observed between the obtained and the expected sequences. The analysis of the results showed that the errors preexisted the rearraying. Using robots for clone handling and bare-codes for microplate tracking during the rearraying procedure allowed us to keep a low error rate. Secondly, the quality control of the PCR amplification showed 6% empty wells and 0.3% double bands. The spots corresponding to the double bands samples were flagged and eliminated in the subsequent microarray data analysis. Finally, the microarrays were controlled by oligonucleotide hybridisation. One negative control was positioned at the end of each block to check the absence of cross-contamination during the spotting (Figure 4). The median signal of these negative spots was used to calculate the general background and was compared to the signal of each spot. A spot was stated as present if its signal was superior to a threshold of 3 times background signal. A microarray batch was validated if 95% of expected spots were present and 100% of negative control spots were absent. In a previous analysis (data not shown), we have observed that the membrane position in the robot has a slight effect on the spotting quality. Then, we systematically proceeded to a hybridisation control on 2 extreme microarrays of each robot's tray. The microarrays batch was validated if all controlled micromembranes of this batch were validated. To date, about 1000 valid micromembranes were produced.

This nylon array is a valuable tool for transcriptome analysis. The use of radiolabelled complex probes allows to detect low-expressed mRNAs using small total RNA amounts (about 100 ng of total RNA) [38]. Such arrays have been used in several studies on human cancer [39] trout reproduction [40] and pig transcriptome [24], Rigaldie, E and Liaubet, L personal communications).

#### Microarray Gene Ontology

**Figure 5:**
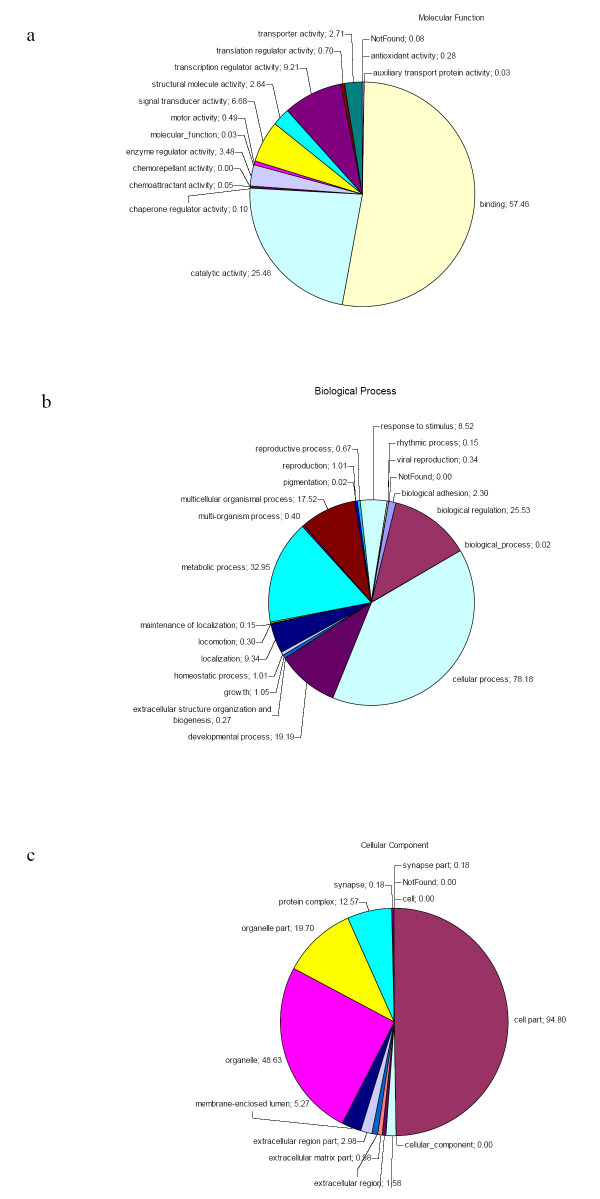
**Gene Ontology annotation of the generic 9K pig microarray**. 5.1, 5.2 and 5.3 indicate the distribution of the annotated contigs into molecular function, biological process and cellular component, respectively.

Gene Ontology annotation was performed through a blastx strategy, against Swissprot protein database. The recovery of a GO annotation was better for the pig generic microarray (70.6%) than for the all assembly contigs (34.4%). This can be explained by the poorer GO annotation of the 54653 singletons of the all assembly (21%), that were not chosen in the microarray design. The frequencies of the major GO categories were about the same for the generic array versus the all assembly: 32.6% (vs 33%) for biological process, 29.8% (vs 29.8%) for cellular component and 37.6% (vs 37.2%) for molecular function. The frequencies of the subcategories were calculated and are displayed in Figure 5. Chi-square test, performed on the subcategory GO frequencies showed that the 9K-microarray was a good representation of the available pig public sequences. It can therefore be used without bias to undertake transcriptome analysis on any model or function.

## Conclusion

We constructed a pig multi-tissue cDNA library which has been successfully normalised and subtracted. This library is derived from the most diverse tissue representation to date. It provides a large set of clones, with limited redundancy but good representation of the complex set of initial tissues. The 24,449 sequences allowed a precise characterisation of the library and contributed to international cDNA sequencing effort.

The 9K nylon cDNA microarray is now used in several gene expression profiling projects, in pig health, reproduction and meat quality.

## Methods

### Tissue collection and RNA preparation

Research involving animal experimentation is approved and controlled by INRA (Institut National de la Recherche Agronomique) (authorisation B-35-275-32 and A37801). Animals were either reared at UE967 Génétique expérimentale en productions animales in Le Magneraud (France) and slaughtered at the Unité Mixte de Recherche SENAH in Saint Gilles (France) or reared and slaughtered at Unité Pluri-Espèces d'Expérimentation Animale in Tours-Nouzilly (France). Forty-four tissue samples from Meishan and Large White pigs at different stages of development or in different physiological conditions (foetus, young or adult animal, male or female, pregnant, stressed or control animals) were taken, frozen in liquid nitrogen and stored at -80°C until RNA extraction.

Total RNA was extracted, using the Chomczynski method [41] and controlled (integrity, reverse transcription efficiency) resulting in 38 high quality preparations.

These total RNA were pooled into 6 groups in equal proportions, according to biological functions (Table 1): brain, digestive function, glands, heart and muscle, male reproduction, female reproduction. PolyA+ mRNA was extracted from 300 μg of RNA from these pools.

As a control, 3 exogenous polyA+ mRNAs ("spikes") obtained by *in vitro *transcription of the corresponding cDNA sequences of SRG3 (*A. thaliana*, X98376), luciferase (*P. pyralis*, CVU03687) and I11a (*A. thaliana*, Y10291) were added to the polyA+ RNAs of each library in different amounts: 6.578 ng, 104.15 pg and 0.274 pg/μg pig RNA respectively. These concentrations correspond to the respective estimated frequencies of 0.5, 50 and 5000 copies of mRNA per cell.

### Library construction, normalisation and subtraction

The libraries were constructed, normalised and subtracted following the protocol of Soares [42] with minor modifications. Briefly, 1 μg of polyA+ RNA (including the 3 spikes) from each pool was used and the reverse transcription with Superscript II (Invitrogen) was primed with 1 μg of NotI-Tag-dT18 primer (see Additional file 2), containing the sequence AGCAG as a library tag. Second-strand synthesis was performed with T4 DNA polymerase (Biolabs) in the presence of DNA ligase (Biolabs) and RNase H (Amersham Pharmacia biotech). cDNA were size-selected (>500 bp), using a BioGel A 50 (BioRad) gel filtration, ligated to EcoRI adaptators primer (see Additional file 2) (Amersham Pharmacia biotech) and digested with *NotI*. The purified cDNAs were directionally cloned into a pT3T7-pac vector and electroporated into DH10B *E coli *bacteria. The number of recombinant clones was determined, for each library, by dilution titration of bacteria onto ampicillin plates. The whole six libraries were pooled and the resulting library was normalised.

The normalisation was achieved through the reassociation of an excess of cDNA inserts, amplified by PCR, with single-stranded plasmid circles, obtained from the starting library (I) [42]. Single-stranded plasmids were generated *in vivo *and purified by chromatography on hydroxyapptite (HAP). One ng of the single-stranded library was used in a high-fidelity PCR (Qiagen Taq Polymerase, 250 UI, reference 201203) with T3 and T7 primers primer (see Additional file 2). 500 ng of PCR products were mixed with 50 ng of the single-stranded library and allowed to hybridise for 22 hours (Cot = 5). The remaining single-stranded circles were purified by HAP chromatography, converted into double-stranded plasmids with T7 sequenase (USB, reference 707752), and electroporated into DH10B bacteria. This led to the normalised library (N) The bound double-stranded fraction was recovered from the HAP column and used to generate a mini-library (A) enriched for abundant mRNAs.

The rate of empty clones and the average size of the inserts were estimated by a PCR amplification of the inserts from 96 clones, by using primers (M13/24 and M13Raster, see Additional file 2) flanking the vector-cloning sites primer.

The subtraction was achieved in a similar way using a Cot = 50: 50 ng of the single-stranded normalised library was reassociated with 2.5 μg of PCR products (primers M13/24 and M13Raster, see Additional file 2) generated from the 8736 sequenced clones of the normalised library. In order to eliminate one over-represented contig, 10% of these PCR products were generated from 4 clones chosen to represent the consensus sequence of this contig.

The quality of the normalisation or subtraction was assessed using the external controls in southern blot and PCR experiments. For southern blot experiment, 500 ng of the I, A and N libraries were separated by electrophoresis on a 1% agarose gel and blotted onto a nylon membrane. The blot was hybridised with a labelled probe corresponding to the external controls. PCR experiments were done with specific primers for external controls (see Additional file 2): 30 cycles of amplification were performed, using different amounts of plasmid DNA from each library (A, I or N) as a template. The PCR products were analysed on 1% agarose gel. The sequencing of 96 to 384 randomly picked clones was also used to assess the quality of the normalisation or subtraction processes.

### EST sequencing

The recombinant bacteria were plated onto 2YT/ampicillin plates and picked into 96 or 384-well plates using a BioPick (Génopole de Toulouse[43]) or a QPix (CRB-GADIE [44]) robot and grown in 10% glycerol medium. Four copies of the plates were made and stored at -80°C. Control plates were generated by picking 2 or 8 (96 or 384-well plates) clones from each sequencing plate. They were also sequenced and used as a sequence-quality control.

A total of 23712 clones were sequenced, following plasmid DNA preparation, from either 5' or both ends by MilleGen^® ^Biotechnologies [45] using M13 (-43) or M13R (-47) primers (see Additional file 2) andBigDye V3.1 (Applied Biosystem) or ET terminator (Amersham) chemistries.

### Sequence analysis and clustering

The data files produced by MilleGen^® ^Biotechnologies were processed by SIGENAE and the documentation on the procedures is available on SIGENAE website [30]. Briefly, the sequences were cleaned up from vector and adaptator sequences; repeats and contaminants were removed by comparison with several sequence databases: Univec, Yeast and E. coli genomes as well as pig ribosomal and mitochondrial genomes. Exogenous control sequences were also removed. PolyA site was identified by its relative position to the vector multiple cloning site and 2 putative polyadenylation sites (AATAAA or ATTAAA) were searched within the 30 bases preceding the polyA site. Valid sequences, that is with a PHRED score over 20 on at least 100 bp, were submitted to the EMBL-EBI Nucleotide Sequence database [28]. All public pig sequences were clustered. Redundancy of the library was calculated as follows: redundancy = 1 - (number of genes/number of clones). The number of genes is estimated by the number of contigs obtained at the end of the SIGENAE processing chain.

The identification and annotation retrieval of the 20 deepest contigs of the assembly and of the 20 deepest AGENAE specific contigs was done by SQL requests on the SIGENAE database. For the deepest contigs, the sequences from Agenae libraries were counted.

Sequences corresponding to putative "tissue specific" proteins in the normalised or subtracted library were identified using a best blast hit strategy for the approximation of ortholog pig genes. The tissue-specificity was documented by literature and the contig containing the nucleotide sequence of the gene referenced in the publication was searched through the SIGENAE WEB interface. If the publication of the sequence was posterior to the SIGENAE assembly, a blastn of the sequence against public_pig_contigV3 database was performed and the contig with a 0 E-value considered. Then an AGENAE sequence was identified in the contig.

### Microarray design, production, quality control and Gene Ontology analysis

#### Design

According to clone availability, the contigs represented by at least one Agenae or USDA clone were selected and the size of the insert was estimated for the different clones.

The following descending order criteria were examined:

- the size of the insert had to be between 0.7 and 1.5 kb-long

- priority was given to a Agenae clone

- priority was given to the longest-insert Agenae clone

Other clones came from home-made libraries and chosen by INRA researchers.

#### Rearraying

The 9138 selected clones have been rearrayed from different libraries. The origin plates were replicated in 384 wells plates in a fresh version for a best result of the subsequent rearraying. These steps were conducted using a Q-Bot robot (Genetix, UK). The bacteria were grown overnight in 2YT (Yeast Tryptone) culture medium containing carbenicilin (100 μg/ml) and glycerol (8%).

To assess the quality of the different steps from sequencing to rearraying, the 4 corners of all plates were controlled by sequencing (4 clones/corner); the obtained sequences were compared with the expected sequences.

#### Amplification

PCR amplifications were performed in 96-well microtiter plates using the u-pig-CRB and l-pig-CRB primers (see Additional file 2), which were specific of the polylinker sequence of vectors used (pCMVSPORT6 for USDA libraries and pT7T3D-pac, pbluescript, pCR 2.1-topo, pUC for INRA libraries). The reactions were performed by transferring 4 μl of *Escherichia coli *in growth culture to 100 μl of PCR mix, containing 1.5 mM MgCl_2_, 1 M betaine, 100 μM dATP, dTTP, dGTP, and dCTP, 1× Promega buffer and 5 U of *Taq *polymerase (M1865, Promega, Madison, WI). All the steps were conducted by a RapidPlate liquid-handling machine (Caliper LifeSciences). The plates were incubated for 3 min at 94°C, before 35 cycles of 94°C for 30 s, 60°C for 30 s and 72°C for 120 s. Amplification products were not quantified, but their quality was systematically checked on 1% agarose gels.

#### Spotting

unpurified PCR products were evaporated, resuspended in 20 μl of distilled water, then transferred to 384-well microplates and spotted ontonylon membranes (Hybond-N+; Amersham Biosciences, Saclay, France), using a Biorobotics MicroGrid-II arrayer (Genomics Solution, Cambridge, U.K.) equipped with a 64-pins Bioroboticsprinthead and 64 Biorobotics 100 μm solid pins. The spotted DNA was denaturated in 150 mM NaOH, 1.5 M NaCl, neutralised in 1 M Tris HCl (pH 7.5), 1.5 M NaCl. After rinsing micromembranes in 2× SSC, the DNA was fixed by successive heat (80°C during 2 hours) and UV (120000 μJ) treatments.

#### Quality control

to control the quality of the nylon microarrays, a vector probe hybridisation (5'-TCACACAGGAAACAGCTATGAC-3')was performed (as described in ) on 8% of the micromembranes.

#### Gene Ontology analysis

The consensus sequence of all the contigs were blasted (blastx, e-value < 10-5) against SwissProt database (version 48). The Gene Ontology annotations were recovered from the best swissprot hit. The proportion of annotated contigs and the proportion of each GO term category was calculated for 2 data sets: 9K microarray contigs and all SIGENAE contigs. A chi-square test (p-value < 0.001) was performed to test if the microarray was enriched in particular GO terms.

## Authors' contributions

AB carried out the library construction, normalisation and subtraction. EI performed the sequence processing, assembly and the gene ontology annotation and assisted the array design. KH carried out the clone rearrangement, PCR amplification, array spotting and quality control. FB participated in the clone picking and arraying and gave technical assistance for the robotic management. MBS and MFB welcomed AB in their laboratory and helped her for the construction of the library. FH initiated the study and supervised the experiments. GTK performed the design of the library, the preliminary experiments for library construction, the sequence data analysis, the array design and wrote the manuscript. All authors read and approved the final manuscript.

## Supplementary Material

Additional file 1 Sequence accession numbers. The accession numbers of 24449 published sequences are listed. Click here for file

Additional file 2 Primer sequences. The name and the sequence of the primers are listed. Click here for file
