# Supplementary material for: A pig multi-tissue normalised cDNA library: large-scale sequencing, cluster analysis and 9K micro-array resource generation
Source: BMC Genomics. 2008 Jan 14;9:17. doi: 10.1186/1471-2164-9-17 (PMC2257943; doi:10.1186/1471-2164-9-17)
Supplement: Additional file 1 — Sequence accession numbers. The accession numbers of 24449 published sequences are listed. [file 1471-2164-9-17-S1.doc]

BX664756-64,BX664767-78,BX664781-815,BX664817-20,BX664822-3,BX664828-77,BX664880-6,BX664889-95,BX664897,BX664899-5011,BX665014-30,BX665032-6,BX665038,BX665041-149,BX665151-8,BX665160-6,BX665168-79,BX665181-202,BX665204-37,BX665240-2,BX665244-6,BX665248-9,BX665251-65,BX665267-96,BX665298-301,BX665303-12,BX665314-23,BX665326-65,BX665368-71,BX665373-80,BX665382-4,BX665387-96,BX665399-419,BX665422-97,BX665500-1,BX665503-29,BX665537-63,BX665566-79,BX665582-92,BX665594-642,BX665646-52,BX665655-68,BX665670,BX665672-84,BX665686-748,BX665751-4,BX665756,BX665758,BX665760-2,BX665764-76,BX665778,BX665780-92,BX665794-818,BX665820-8,BX665830-3,BX665835-9,BX665842-4,BX665846-55,BX665857-8,BX665860-2,BX665864-5,BX665867-75,BX665877-9,BX665881-7,BX665889-95,BX665897,BX665899-902,BX665904-10,BX665912-8,BX665920-1,BX665923-9,BX665931-57,BX665960-1,BX666067-131,BX666133-240,BX666242-4,BX666246-73,BX666275-300,BX666302-6,BX666308-75,BX666377,BX666379-431,BX666433-60,BX666462-79,BX666481-539,BX666541-64,BX666566-71,BX666573-610,BX666612-24,BX666626-38,BX666640-4,BX666646-55,BX666657-8,BX666660-71,BX666673-88,BX666690-728,BX666730-55,BX666757-87,BX666789-95,BX666797-807,BX666809-45,BX666847-79,BX666881-90,BX666892-3,BX666895-903,BX666905-91,BX666993-5,BX666997-7075,BX667078-90,BX667092-130,BX667132-55,BX667157-84,BX667186-93,BX667195-220,BX667222-4,BX667226-53,BX667255-307,BX667309-15,BX667318-9,BX667321-37,BX667339-69,BX667371-92,BX667394-414,BX667416-52,BX667454-524,BX667526,BX667597-607,BX667610-21,BX667623-38,BX667640-4,BX667646-71,BX667673-89,BX667691-711,BX667713-32,BX667734-6,BX667738-41,BX667743-51,BX667753-74,BX667776-800,BX667802-22,BX667824-36,BX667838-49,BX667851-4,BX667856,BX667858-85,BX667887-93,BX667895-902,BX667904-23,BX667925-35,BX667937-43,BX667945-8,BX667950-62,BX667964-75,BX667977-8004,BX668006-24,BX668026-36,BX668038-66,BX668068-76,BX668078-84,BX668086-94,BX668096-129,BX668131-2,BX668134-50,BX668152-4,BX668156-60,BX668162-8,BX668170-6,BX668178-9,BX668181-6,BX668188-93,BX668195-7,BX668200-7,BX668209-25,BX668228-40,BX668242-8,BX668250-79,BX668281-7,BX668289-308,BX668310-3,BX668315-9,BX668321-51,BX668353-9,BX668361-3,BX668365-75,BX668377-9,BX668381,BX668383-90,BX668392-410,BX668412-6,BX668418-21,BX668424-31,BX668433-44,BX668446-51,BX668453,BX668455-8,BX668460-1,BX668463-91,BX668494,BX668496,BX668498-524,BX668527,BX668530-45,BX668547-51,BX668553-607,BX668610-34,BX668636-54,BX668656-704,BX668706-15,BX668717-35,BX668737-55,BX668758-64,BX668766-8,BX668770-80,BX668782,BX668785-99,BX668801-73,BX668875-907,BX668909-72,BX668975-98,BX669000-36,BX669038-45,BX669047-72,BX669074-177,BX669179-83,BX669185-226,BX669228-55,BX669257,BX669260,BX669263-4,BX669266-73,BX669275-301,BX669304-11,BX669313-28,BX669331-2,BX669334-49,BX669351-9,BX669361-73,BX669375-85,BX669387,BX669389-92,BX669394-5,BX669398-413,BX669417-21,BX669423-51,BX669453-63,BX669465-96,BX669499-567,BX669569-601,BX669603-33,BX669635-52,BX669654-702,BX669704-29,BX669731,BX669734-65,BX669767-75,BX669777-80,BX669782-3,BX669786-817,BX669819-65,BX669867-95,BX669897-8,BX669900-8,BX669911-8,BX669920-36,BX669938-62,BX669964-88,BX669990-70049,BX670051-73,BX670076-81,BX670084-114,BX670116-23,BX670126-45,BX670147-66,BX670173-82,BX670184-231,BX670233-96,BX670298-305,BX670308-15,BX670318-31,BX670333-46,BX670348,BX670350-4,BX670358-61,BX670363,BX670366-96,BX670398-414,BX670416-76,BX670478-80,BX670482-4,BX670486-90,BX670492-504,BX670506-49,BX670552-73,BX670575-87,BX670589-97,BX670599-606,BX670610-1,BX670613-71,BX670673-8,BX670680-731,BX670733-70,BX670772-82,BX670784-800,BX670803-17,BX670820-49,BX670851-911,BX670914,BX670917-58,BX670963-86,BX670988-1032,BX671034-58,BX671060-3,BX671065-102,BX671105-22,BX671124-31,BX671133-44,BX671146-79,BX671181-209,BX671214-41,BX671243-57,BX671260-7,BX671269-71,BX671274-89,BX671291,BX671293-322,BX671324-34,BX671336-98,BX671400-30,BX671433-9,BX671441-4,BX671446-7,BX671450-71,BX671473-4,BX671476-80,BX671482-576,BX671578-602,BX671604-80,BX671683-706,BX671709-28,BX671731-90,BX671792,BX671794-807,BX671809,BX671811-66,BX671868-85,BX671887-97,BX671900-46,BX671948-59,BX671961-84,BX671987-2007,BX672009-35,BX672038-9,BX672042-3,BX672045-7,BX672049-67,BX672069-79,BX672081-93,BX672095-6,BX672098-124,BX672126-55,BX672157-83,BX672185-257,BX672259-304,BX672306-11,BX672313-72,BX672374-422,BX672424-47,BX672449-512,BX672514-30,BX672533,BX672535-6,BX672538-65,BX672567-89,BX672591-636,BX672638-56,BX672658-9,BX672661-94,BX672696-705,BX672707-10,BX672712-5,BX672717-8,BX672721-9,BX672731-6,BX672738-71,BX672773,BX672775-83,BX672785-806,BX672808-15,BX672817-9,BX672821-5,BX672827-34,BX672836-40,BX672842-59,BX672861-79,BX672881-8,BX672890-907,BX672909-23,BX672925-8,BX672930-3010,BX673012-4,BX673017-24,BX673026-71,BX673073-4,BX673076-108,BX673110-62,BX673164,BX673166-74,BX673176-225,BX673227-38,BX673240-8,BX673250-2,BX673254-83,BX673285-308,BX673311-6,BX673318-58,BX673360-72,BX673374-6,BX673378-412,BX673414-7,BX673419-30,BX673432,BX673434-50,BX673452-6,BX673458-74,BX673476-509,BX673511-3,BX673515-20,BX673522-46,BX673548-58,BX673560-82,BX673584-628,BX673631-45,BX673647-52,BX673654-63,BX673665-9,BX673671,BX673673-700,BX673702-17,BX673719-35,BX673737-47,BX673749-78,BX673780-6,BX673788-90,BX673792-814,BX673816-31,BX673833-43,BX673846,BX673850-70,BX673872-95,BX673898-922,BX673924-6,BX673930-42,BX673944-63,BX673965-85,BX673988-9,BX673991-4005,BX674007-13,BX674015-9,BX674021-36,BX674038-46,BX674048-68,BX674070-107,BX674109-27,BX674129-31,BX674133-61,BX674163-213,BX674215-21,BX674223-33,BX674235-63,BX674266-82,BX674284,BX674286-92,BX674294-5,BX674297-305,BX674307-12,BX674314-8,BX674320-1,BX674323-34,BX674336-68,BX674370-6,BX674378-86,BX674388-99,BX674401-35,BX674437-66,BX674468-76,BX674478-84,BX674486-533,BX674535-54,BX674556-7,BX674560-8,BX674570-629,BX674631-41,BX674643-5,BX674647-59,BX674661-7,BX674669-75,BX674677-86,BX674688-701,BX674703-6,BX674708-9,BX674711-9,BX674721-47,BX674749-67,BX674769-75,BX674777-84,BX674786-96,BX674798-842,BX674844-9,BX674851-6,BX674858-62,BX674864-74,BX674876-909,BX674911-25,BX674927-88,BX674990-5012,BX675014,BX675016-26,BX675028-9,BX675031-94,BX675096-107,BX675111-34,BX675136-41,BX675201-4,BX675206-65,BX675267-300,BX675302-46,BX675348-54,BX675356-63,BX675365-456,BX675458-76,BX675478-85,BX675487-90,BX675492-7,BX675499-526,BX675528-82,BX675584-667,BX675669-818,BX675820-5,BX675827-43,BX675845-970,BX675972-83,BX675985-6038,BX676040-103,BX676106-19,BX676122-8,BX676130-44,BX676146-216,BX676218-66,BX676268-72,BX676274-84,BX676286-95,BX676297-309,BX676311-3,BX676315,BX676317,BX676319-53,BX676355-65,BX676367-460,BX676462-72,BX676474-509,BX676511-30,BX676532-4,BX676536-42,BX676544,BX676546-61,BX676563-680,BX676683-733,BX676735-47,BX676749-50,BX676752-69,BX676771-7,BX676779-83,BX677239-64,BX677266-87,BX914224-78,BX914280-334,BX914336-43,BX914345-52,BX914354-562,BX914564-76,BX914578-90,BX914592-661,BX914663-82,BX914684-99,BX914701-29,BX914731-4,BX914736-51,BX914753-75,BX914777-9,BX914781-843,BX914846-92,BX914894-927,BX914929-68,BX914970-86,BX914988-9,BX914991-5067,BX915069-144,BX915146-266,BX915268-314,BX915316-31,BX915333-46,BX915348-87,BX915389-463,BX915465-546,BX915548-99,BX915601-18,BX915620-1,BX915623-44,BX915646-765,BX915767-840,BX915842-56,BX915858-76,BX915878-924,BX915926-55,BX915957,BX915959,BX915961-81,BX915983-6049,BX916051-64,BX916066-184,BX916186-247,BX916249-99,BX916301-4,BX916306-457,BX916459-570,BX916572-6,BX916578-80,BX916583-5,BX916587-628,BX916630-99,BX916701-6,BX916708-34,BX916736-71,BX916773-812,BX916814-24,BX916826-61,BX916863-79,BX916881-904,BX916906-19,BX916921-7,BX916929-73,BX916975-85,BX916987-98,BX917000-9,BX917011-57,BX917059,BX917061-123,BX917125-57,BX917159-77,BX917179-270,BX917272-3,BX917275-313,BX917315-21,BX917323-65,BX917367-84,BX917386-403,BX917405-20,BX917422-71,BX917473-551,BX917553,BX917555-86,BX917588-614,BX917616-39,BX917641-711,BX917713-5,BX917717-800,BX917802-35,BX917837-53,BX917855-927,BX917929-90,BX917992-5,BX917997-8110,BX918112-3,BX918115-53,BX918155-91,BX918193-219,BX918221-5,BX918227-54,BX918256-69,BX918271-300,BX918302-22,BX918324-55,BX918357-416,BX918418-21,BX918423-57,BX918459-67,BX918469-94,BX918496-506,BX918508-98,BX918600-64,BX918667-86,BX918688-712,BX918714-45,BX918747,BX918749-94,BX918796-812,BX918814-54,BX918856-79,BX918881-97,BX918899-911,BX918913-93,BX918995-9094,BX919096-244,BX919246-84,BX919286-324,BX919326-89,BX919391-5,BX919397-453,BX919455-545,BX919548-739,BX919742-804,BX919806-21,BX919823-66,BX919868-73,BX919875-84,BX919886-94,BX919896-910,BX919912-24,BX919926-20028,BX920030-130,BX920132-53,BX920155-94,BX920196-357,BX920359-90,BX920392-400,BX920402-30,BX920432-9,BX920441-4,BX920446-56,BX920458,BX920460-79,BX920481-542,BX920544-54,BX920556-69,BX920571-717,BX920719-27,BX920729-30,BX920732-73,BX920775-805,BX920807-16,BX920818-66,BX920868-91,BX920893-1016,BX921018-132,BX921134-5,BX921137-67,BX921169-71,BX921173-247,BX921249-70,BX921272-302,BX921304-47,BX921349-50,BX921352-442,BX921444-61,BX921463-512,BX921514-57,BX921559-680,BX921682-705,BX921707-14,BX921716-41,BX921743-55,BX921757-804,BX921806-57,BX921859-924,BX921926-49,BX921951-2001,BX922003-34,BX922036-46,BX922048-94,BX922096-235,BX922237-96,BX922298-402,BX922404-50,BX922452-99,BX922501-23,BX922525-83,BX922585-724,BX922726-50,BX922752-820,BX922822-51,BX922853-928,BX922930-66,BX922968-3048,BX923050-61,BX923063,BX923065-84,BX923086-164,BX923166-78,BX923180-203,BX923205-21,BX923223-66,BX923268-81,BX923283-356,BX923358-420,BX923422-500,BX923502-9,BX923511-45,BX923547-62,BX923564-727,BX923729-45,BX923747-53,BX923755-825,BX923827-46,BX923848-55,BX923857-61,BX923863-72,BX923874-85,BX923887-958,BX923960-71,BX923973-4,BX923976-4014,BX924016-122,BX924124-31,BX924133-7,BX924139-258,BX924260-8,BX924270-396,BX924398-406,BX924408-28,BX924430-1,BX924433-528,BX924530-5,BX924537-44,BX924546-52,BX924555-76,BX924578-97,BX924599-660,BX924662-745,BX924747-76,BX924778-848,BX924850-67,BX924869-905,BX924907-32,BX924934-68,BX924970-81,BX924983-96,BX924998-5019,BX925021-53,BX925055-60,BX925062-76,BX925078-91,BX925093-170,BX925172-317,BX925319-46,BX925348-50,BX925352-94,BX925396-484,BX925488-519,BX925521-636,BX925638-48,BX925650-71,BX925673-7,BX925679,BX925681-716,BX925718-833,BX925835,BX925837-911,BX925913-44,BX925946-86,BX925988-6041,BX926043-130,BX926132-332,BX926334-420,BX926422-48,BX926450-554,BX926556-66,BX926568-695,BX926697-706,BX926708-17,BX926719-28,BX926730-97,BX926799-811,BX926813-51,BX926853-84,BX926886-7019,BX927021-61,CT971555-9,CT971570-1
