# Supplementary material for: A pig multi-tissue normalised cDNA library: large-scale sequencing, cluster analysis and 9K micro-array resource generation
Source: BMC Genomics. 2008 Jan 14;9:17. doi: 10.1186/1471-2164-9-17 (PMC2257943; doi:10.1186/1471-2164-9-17)
Supplement: Additional file 2 — Primer sequences. The name and the sequence of the primers are listed. [file 1471-2164-9-17-S2.doc]

| Primer | Sequence |
| --- | --- |
| NotI-Tag-dT18 | TGT TAC CAA TCT GAA GTG GGA GCG GCC GCA GCA GT_18_ |
| EcoRI adaptator | AAT TCG GCA CGA G |
| T3 | ATT AAC CCT CAC TAA AGG GA |
| T7 | TAA TAC GAC TCA CTA TAG GGA |
| M13/24 | GGT TTT CCC AGT CAC GAC GTT GTA |
| M13Raster | AGC GGA TAA CAA TTT CAC ACA GG |
| uI11a | AAA TGG GAA ATG AGA CGA AGA |
| lI11a | TTG GTG AGA TTG ATG CTT TGA |
| uFFLucif | GGA AGA CGC CAA AAA CAT AA |
| lFFLucif | GCA TAC GAC GAT TCT GTG A |
| uSRG3 | GAT AGC AGC ACC CAC ATT G |
| lSRG3 | GAG GAG ATA ACA GAG GCG G |
| M13 (-43) | AGG GTT TTC CCA GTC ACG ACG TT |
| M13R (-47) | GCG GAT AAC AAT TTC ACA CAG |
| u-pig-CRB | GAGCGGATAACAATTTCACACAGGA |
| l-pig-CRB | TCCCAGTCACGACGTTGTAAAACGA |

| N° library | Tissues  Adult (A), young (Y) or fetal (F) animal | Number of recombinant clones |
| --- | --- | --- |
| 1-Brain | Hippocampus (A)  Hypothalamus (A) Pituitary gland (A)  Cerebral trunk (A)  Brain (F) | 800 000 |
| 2-Digestive function | Stomach (A + F)  Small intestine (A + F)  Large intestine (A + F)  Gall-bladder (A) | 822 500 |
| 3- Glands | Adrenals (A)  Kidney (A)  Liver (A + F)  Thymus (A + Y)  Spleen (A)  Pancreas (A) | 800 000 |
| 4- Heart and muscle | Heart (A + F)  Muscle (A + F)  Skin (A)  Melanocytes (A)  Adipose tissue (A) | 1 800 000 |
| 5-Male reproductive organs | Gonads (F)  Epididymis (A) Seminal vesicle (A) Bulbo uretral gland (A)  Testis (A) | 780 000 |
| 6- Female reproductive organs | Gonads (F)  Ovary (A + F)  Uterus (A)  Placenta  Mammary gland (A) | 1 325 000 |
| Normalized (N) | Mix of libraries 1 to 6 | 6 400 000 |
